# Applicability of Oncotype DX Testing in a Diverse Breast Cancer Population in Hawaii

**DOI:** 10.1155/tbj/9104103

**Published:** 2025-09-01

**Authors:** Kyle Xia, Eileen Chen, Roxana Hu, Ian Pagano, Jami Fukui

**Affiliations:** ^1^University of Hawai'i John A. Burns School of Medicine, Honolulu, Hawaii, USA; ^2^University of Hawai'i John A. Burns School of Medicine Residency Training Program in Obstetrics and Gynecology, Honolulu, Hawaii, USA; ^3^University of Hawai'i Cancer Center, Honolulu, Hawaii, USA

**Keywords:** breast cancer, disparities, Oncotype DX, race

## Abstract

**Purpose:** The Oncotype DX test is standardly used for patients with early-stage, hormone-receptor–positive, HER2-negative breast cancers to determine the benefit from chemotherapy and the likelihood of distant recurrence. The relationship between Oncotype DX recurrence scores and race/ethnicity is still being studied. This retrospective study aims to evaluate the relationship between Oncotype DX recurrence scores, race/ethnicity, and clinicopathological factors and to support the applicability of the Oncotype DX test for a diverse breast cancer population of Hawaii.

**Materials and Methods:** We evaluated 879 breast cancer cases diagnosed from January 2018–March 2022 within a major health system in Hawaii, 600 of which received Oncotype DX recurrence scores to provide prognostic and therapy-predictive information based on NCCN guidelines. Linear regression with both univariable (unadjusted) and multivariable (adjusted for all other variables) models was run on the 600 breast cancer cases that received Oncotype DX recurrence scores. The predictor variables were age at diagnosis, race, tumor size, ER/PR status, and histology.

**Results:** On multivariable analysis, we found statically significant differences in Oncotype DX recurrence scores according to age (60–69 vs. 18–49, *p*=0.01), ER/PR status (PR-positive vs. PR-negative, *p* < 0.0001), histology (other vs. ductal, *p*=0.004), and tumor size (2–5 cm vs. 0-1 cm, *p*=0.0003). We found no significant differences in Oncotype DX recurrence scores according to race/ethnicity.

**Conclusion:** Our findings indicate that Oncotype DX recurrence scores are not variable according to race/ethnicity, highlighting the need for further research to understand the known disparities in breast cancer outcomes among different racial/ethnic groups. Our study supports the correlation between Oncotype DX recurrence scores and other factors and aligns with established prognostic trends for these variables in a diverse population. This study supports the applicability for the Oncotype DX test in a diverse breast cancer population of Hawaii.

## 1. Introduction

Breast cancer incidence and mortality is known to differ across racial/ethnic groups and age. According to the American Cancer Society update in breast cancer statistics in the United States, the incidence of breast cancer was highest at 133.7 new cases per 100,000 people in White patients and 127.8 new cases per 100,000 people in Black patients from 2015 to 2019 [1]. However, the breast cancer mortality rate in 2016–2020 was 40% higher in Black patients compared to White patients [1]. Other studies also suggest a disproportionately high mortality rate for Black, Asian, and Pacific Islander patients compared to White patients in recent years [2]. In Hawaii, the overall incidence of breast cancer is higher than the national average and highest among Japanese and Native Hawaiian patients [3]. In 2014–2018, the breast cancer mortality rate was 82% higher in Native Hawaiians compared to Japanese patients [3].

Although the current research shows disproportionate rates of breast cancer incidence and mortality across racial/ethnic groups, the relationship between race/ethnicity and the Oncotype DX recurrence score is still being studied. For patients with early-stage, hormone-positive, HER2-negative breast cancers, the 21-gene assay (Oncotype DX) provides a recurrence score between 0 and 100 that can be used to estimate the likelihood of breast cancer recurrence and potential benefit of adjuvant chemotherapy. Higher Oncotype DX recurrence scores estimate a higher likelihood of distant breast cancer recurrence and greater benefit of receiving adjuvant chemotherapy. A recent cohort study using the Surveillance, Epidemiology, and End Results (SEER) Oncotype DX 2004–2015 database found that Black women in the United States were more likely to experience breast cancer–specific mortality from ER-positive, HER2-negative, axillary-node–negative breast cancer compared with non-Hispanic White women with similar Oncotype DX recurrence scores, even after adjusting for age, year of diagnosis, tumor size, progesterone receptor status, type of surgery, and administration of radiation therapy and chemotherapy [4]. This suggests that the Oncotype DX recurrence score may have lower prognostic accuracy in Black women and that the Oncotype DX test is just one of the several factors to utilize for prognostication purposes. These findings challenge the generalizability of the Oncotype DX recurrence score in racial/ethnic minority groups, prompting further assessment of the Oncotype DX test in diverse populations that were underrepresented in pivotal studies that established clinical validity and utility of the test.

In our study, we evaluated the relationship between Oncotype DX scores and race/ethnicity, specifically for Asian subpopulations and Native Hawaiians/Pacific Islanders, along with patient demographics and clinicopathological factors, including age at diagnosis, tumor characteristics, ER/PR status, tumor size, and histology. The goal of our study was to evaluate the applicability of the Oncotype DX test for a diverse breast cancer population of Hawaii.

## 2. Materials and Methods

We analyzed 879 unique breast cancer cases diagnosed in January 2018–March 2022 within the major health system in Hawaii, 600 of which received Oncotype DX recurrence scores to provide prognostic and therapy-predictive information based on NCCN guidelines. We ran linear regression on the Oncotype DX recurrence scores as score ranges (Table 1) and as means (Table 2), using age at diagnosis, race, tumor size, ER/PR status, and histology as predictor variables. Score ranges were based on the Oncotype DX report breakdown, established by pilot studies for the test. We ran both univariable (unadjusted) and multivariable (adjusted for all other variables) models. Age at diagnosis of 18–49 years old, tumor size of 0–10 mm, ER-positive/PR-negative status, ductal carcinoma histology, and White race were established as baselines to which other groups within the same clinicopathological feature were compared. All confidence intervals (CI) are reported as 95% CI.

## 3. Results

From a total of 879 unique breast cancer cases, 600 cases received an Oncotype DX recurrence score to provide prognostic and therapy-predictive information based on NCCN guidelines. In the cases that received an Oncotype DX recurrence score, age of diagnosis ranged from 24 to 89 (mean = 59.6), race/ethnicity included Japanese (*n* = 157, 26.2%), White (*n* = 153, 25.5%), Hawaiian (*n* = 105, 17.5%), Filipino (*n* = 100, 16.7%), Chinese (*n* = 35, 5.8%), and others (*n* = 49, 8.2%). Patients aged 60–69 years (*n* = 205, 34.2%) had a significantly lower adjusted mean Oncotype DX recurrence score of 14.5 (CI = 13.3–15.7,*p*=0.01) compared to patients aged 18–49 years (*n* = 125, 20.8%). Patients with larger tumor sizes were significantly associated with higher Oncotype DX recurrence scores (*p*=0.0005). Cases with tumor size 21–50 mm (*n* = 144, 24.0%) were found to have an adjusted mean Oncotype DX recurrence score of 18.2 (CI = 16.7–19.7, *p*=0.006) which was significantly higher compared to cases with tumor size of 0–10 mm (*n* = 128, 21.3%). Cases with histology other than ductal and/or lobular (*n* = 7, 1.2%), including micropapillary, papillary, or mucinous breast cancer, had an adjusted mean of 9.9 (CI = 4.5–15.4, *p*=0.03) which were statistically significant for a lower Oncotype DX recurrence score compared to ductal histology (*n* = 509, 84.8%). Patients with PR-positive status (*n* = 550, 91.7%) were found to have a mean adjusted Oncotype DX recurrence score of 15.1 (CI = 14.4–15.8, *p* < 0.0001), which was significantly lower compared to patients with PR-negative status (*n* = 50, 8.3%). Patients with PR-negative status were also significantly associated with higher Oncotype DX recurrence scores (*p* ≤ 0.0001). There were no significant differences in Oncotype DX recurrence score by race/ethnicity compared to White patients.

## 4. Discussion

Overall, the patient demographics we studied had variable significance with Oncotype DX recurrence scores. The breast cancer cases with a tumor size of 21–50 mm and PR-negative status correlated with a significantly higher Oncotype DX recurrence score when compared to tumor size of 10 mm or less and PR-positive status, respectively. Moreover, patients with larger tumor sizes and PR-positive status were found with higher Oncotype DX recurrence score ranges. Conversely, cases with age of diagnosis from 60–69 years and tumor histology other than ductal or lobular, including micropapillary, papillary, or mucinous breast cancer, had a significantly lower Oncotype DX recurrence score compared to age of diagnosis from age 18–49 years and ductal or lobular breast cancer, respectively. We also found that race/ethnicity did not appear to correlate with Oncotype DX recurrence scores when we adjusted for age, tumor size, ER/PR status, and histology in our study.

Although tumor size is a well-accepted prognostic factor for breast cancer [5], the literature regarding the relationship between tumor size and Oncotype DX is sparse. One study found that tumor size individually correlates significantly with Oncotype DX recurrence score, but when taken together with grade and Ki-67, it cannot reliably predict the Oncotype DX recurrence score [6]. Some studies found that increasing Oncotype DX recurrence score may not be associated with larger tumor size, which conflicts with our findings [7, 8]. While larger tumors are generally associated with a poorer prognosis and may need more aggressive treatment, one study showed a negative correlation between tumor size and Oncotype DX recurrence score in the PR-negative cases, suggesting that a large tumor with PR negativity that has not metastasized may have favorable tumor biology [8]. Furthermore, studies have shown that PR negativity has an inverse relationship to Oncotype DX recurrence score, which is corroborated by our findings [7]. Regarding age of diagnosis, previous findings have been consistent with our results, showing that younger age of diagnosis is associated with high-risk Oncotype DX recurrence scores [9]. Studies have also shown that tumor histology consistent with micropapillary, papillary, or mucinous breast cancers are associated with a favorable prognosis [10], correlating with our findings of lower Oncotype DX recurrence scores in cases with these tumor histologies.

The current literature examining the relationship between race/ethnicity and Oncotype DX recurrence scores have shown mixed results. Some previous studies have found that Black women are more likely to have a high-risk Oncotype DX recurrence score [11], while other studies have found no significant relationships between race/ethnicity and Oncotype DX recurrence score [12]. One recent cohort study found that Black women in the United States were more likely to have a high-risk Oncotype DX recurrence score as well as a higher mortality compared to non-Hispanic White women with similar scores, suggesting that the Oncotype DX recurrence score may have lower prognostic accuracy in Black women [4]. Although one study validated the significant prognostic value of the Oncotype DX recurrence score in 200 Japanese patients with breast cancer [13], another study found that the predictive value of the Oncotype DX recurrence score for assessing chemotherapy benefit was not validated in non-White women and may lead to over- or undertreatment with adjuvant chemotherapy [14].

Studies that contributed significantly to the development of the Oncotype DX scoring system have a lack of race/ethnicity data. The TailorRx study did not factor in race/ethnicity in their study design. Although the PonderRx study included race/ethnicity in its supplementary appendix with a sample distribution representative of US census data, Asian is considered one racial group. In most of the literature that analyzes the relationship between Oncotype DX recurrence score and race/ethnicity, Asian and Native Hawaiian/Pacific Islander populations are often grouped together as Asian/Pacific Islander. Previous research has shown that aggregating data for Asian, Native Hawaiian, and other Pacific Islander populations can mask health disparities, especially for Native Hawaiian and other Pacific Islander patients [15]. This lack of disaggregated race/ethnicity data leaves a significant gap in knowledge regarding the prognostic and treatment-predictive accuracy of the Oncotype DX scoring system for Asian subpopulations. One study found that Native Hawaiian/Pacific Islander women with early-stage breast cancer had a significantly lower overall survival rate compared to non-Hispanic White women, while all subpopulations of Asian Americans studied had higher survival rate compared to non-Hispanic White women [16]. The disproportionate relationship between breast cancer incidence and mortality by race/ethnicity is not currently explained by Oncotype DX recurrence scores.

Strengths of this study include disaggregation of data for Asian/Pacific Islander and a sample that is more representative of the racial/ethnic diversity in Hawaii, allowing for better generalizability to the Hawaiian patient population compared to the national data. In addition, use of a multivariable model accounts for some confounding variables and reveals true disparities between groups. Our study contributes more information about Oncotype DX recurrence scores within Asian, Pacific Islander, and Native Hawaiian subpopulations.

One limitation of this study includes confounding factors that were not accounted for in our multivariable model. These confounding variables include but are not limited to comorbidities and socioeconomic factors. In addition, the small sample size of our study likely lowers the statistical power of our findings. This is particularly evident with our finding that cases with micropapillary, papillary, or mucinous breast cancers histology have a lower Oncotype DX recurrence score compared to ductal histology, as only 7 cases from our analysis fit these rarer diagnostic criteria. Although data were collected from a major hospital system in Hawaii, this study is subject to selection bias because it only includes a single registry. Another limitation of this study is that patients who received an Oncotype DX recurrence score were not followed longitudinally. This means that data on recurrence and mortality differences between groups and the prognostic accuracy of the Oncotype DX test for this diverse breast cancer population were not assessed.

Our findings highlight the factors that correlate with higher risk Oncotype DX recurrence scores. For example, a breast cancer patient that presents with younger age at diagnosis, larger tumor size, PR-negative status, and ductal or lobular histology will likely have a high Oncotype DX recurrence score. Identification of these factors can help guide development of personalized treatment regimens and early conversations with patients regarding the need for adjuvant chemotherapy to prevent late distant recurrence.

## 5. Conclusions

In summary, our findings suggested no significant correlation between Oncotype DX recurrence scores and race/ethnicity. Further research is required to account for biologic, socioeconomic, and healthcare access factors not included in this study, inorder to explain disparities in incidence and mortality of breast cancer across different races/ethnicities. Further studies can also explore the utility of the Oncotype DX recurrence scores for node-positive breast cancer in the diverse breast cancer population of Hawaii. The relationships between Oncotype DX recurrence and other factors such as age, tumor size, ER/PR status, and histology are found in our study to be consistent with prognostic trends seen for those factors.

## Figures and Tables

**Table 1:** Oncotype DX recurrence score ranges by age, race/ethnicity, tumor size, and histology.

Variable	Value	Total		Low (0-17)		Mid (18–25)		High (26+)		*p*
		*N*	%	*N*	%	*N*	%	*N*	%	
Age	18–49	125	20.8	72	19.1	41	28.3	12	15.2	0.09
	50–59	150	25.0	89	23.7	37	25.5	24	30.4	
	60–69	205	34.2	141	37.5	40	27.6	24	30.4	
	70+	120	20.0	74	19.7	27	18.6	19	24.1	

Race	Chinese	36	6.0	18	4.8	8	5.5	10	12.7	0.43
	Filipino	100	16.7	60	16.0	27	18.6	13	16.5	
	Hawaiian	105	17.5	69	18.4	23	15.9	13	16.5	
	Japanese	157	26.2	98	26.1	41	28.3	18	22.8	
	White	153	25.5	96	25.5	36	24.8	21	26.6	
	Others	49	8.2	35	9.3	10	6.9	4	5.1	

Tumor size (mm)	0–10	128	21.3	87	23.1	26	17.9	15	19.0	0.0005
	11–20	307	51.2	203	54	77	53.1	27	34.2	
	21–50	144	24.0	74	19.7	35	24.1	35	44.3	
	51+	21	3.5	12	3.2	7	4.8	2	2.5	

ER/PR status	+/+	550	91.7	361	96.0	127	87.6	62	78.5	< 0.0001
	+/−	50	8.3	15	4.0	18	12.4	17	21.5	

Histology	Ductal	509	84.8	318	84.6	118	81.4	73	92.4	0.11
	Ductal/lobular	17	2.8	9	2.4	7	4.8	1	1.3	
	Lobular	67	11.2	42	11.2	20	13.8	5	6.3	
	Others	7	1.2	7	1.9	0	0	0	0	

**Table 2:** Oncotype DX recurrence score averages by age, race/ethnicity, tumor size, and histology.

Variable	Value	*N*	%	Univariable				Multivariable			
				Mean	LCL	UCL	*p*	Mean	LCL	UCL	*p*
Age	18–49	125	21	16.8	15.2	18.4		17.0	15.5	18.6	
	50–59	150	25	16.9	15.4	18.4	0.92	17.3	15.9	18.7	0.83
	60–69	205	34	14.8	15.4	18.4	0.06	14.5	13.3	15.7	0.01
	70+	120	20	15.6	13.9	17.2	0.29	15.3	13.7	16.9	0.12

Race	Chinese	36	6	18.5	15.5	21.5	0.11	18.0	15.2	20.9	0.17
	Filipino	100	17	16.0	14.2	17.8	0.82	15.9	14.2	17.6	0.94
	Hawaiian	105	18	15.5	13.7	17.3	0.84	15.6	13.9	17.3	0.88
	Japanese	157	26	16.1	14.7	17.6	0.72	16.1	14.8	17.5	0.74
	White	153	26	15.7	14.3	17.2		15.8	14.4	17.2	
	Others	49	8	14.4	11.8	17.0	0.37	14.6	12.2	17.0	0.41

Tumor size (mm)	0–10	128	21	15.2	13.6	16.8		14.9	13.4	16.4	
	10–20	307	51	15.1	14.0	16.1	0.90	14.8	13.8	15.7	0.86
	20–50	144	24	18.1	16.6	19.6	0.009	18.8	17.4	20.2	0.0003
	50+	21	4	17.8	13.9	21.7	0.22	18.7	14.9	22.5	0.07

ER/PR status	+/+	550	92	15.2	14.4	15.9	< 0.0001	15.1	14.4	15.8	< 0.0001
	+/−	50	8	24.0	21.5	26.5		24.9	22.4	27.3	

Histology	Ductal	509	85	16.0	15.2	16.8		16.0	15.3	16.8	
	Ductal/lobular	17	3	16.9	12.6	21.3	0.67	18.1	13.9	22.2	0.34
	Lobular	67	11.2	16.1	13.9	18.3	0.93	15.3	13.1	17.4	0.51
	Others	7	1.2	5.4	−1.4	12.3	0.003	6.6	0.1	13.0	0.004

## Data Availability

The data are available upon reasonable request by emailing the corresponding author jfukui@cc.hawaii.edu

## References

[1] American Cancer Society (2024). *Breast Cancer Facts and Figures 2022–2024*.

[2] Trentham-Dietz A., Chapman C. H., Bird J., Gangnon R. E. (2021). Recent Changes in the Patterns of Breast Cancer as a Proportion of all Deaths According to Race and Ethnicity. *Epidemiology*.

[3] Hawai’i Tumor Registry (2022). Cancer at a Glance 2014–2018. https://www.uhcancercenter.org/research/shared-resources/hawaii-tumor-registry.

[4] Hoskins K. F., Danciu O. C., Ko N. Y., Calip G. S. (2021). Association of Race/Ethnicity and the 21-Gene Recurrence Score With Breast Cancer-Specific Mortality Among US Women. *JAMA Oncology*.

[5] Giuliano A. E., Edge S. B., Hortobagyi G. N. (2018). Eighth Edition of the AJCC Cancer Staging Manual: Breast Cancer. *Annals of Surgical Oncology*.

[6] Lathrop K. I., Mazo- Canola M., Gelfond J., Shiao J. C., Hinojosa B., Terracina K. A. (2015). Correlation Between Oncotype DX Score With Histologic Grade, Tumor Size and Ki67 in Patients With Early Stage Breast Cancer: A Single Academic Institution Experience. *Journal of Clinical Orthodontics*.

[7] Hanna M. G., Bleiweiss I. J., Nayak A., Jaffer S. (2017). Correlation of Oncotype DX Recurrence Score With Histomorphology and Immunohistochemistry in Over 500 Patients. *International Journal of Breast Cancer*.

[8] Arthur L. E., McMann A. H., Slattery L. N. (2020). Factors That Predict Biological Aggressiveness in Estrogen Receptor-Positive/Human Epidermal Growth Factor Receptor 2-Negative/Lymph Node-Negative Breast Cancer. *The Ochsner Journal*.

[9] Namuche F., Ruiz R. E., Morante Cruz Z. D. (2018). Oncotype Dx Results in Patients ≤ 40 years: Does Age Matter? New Insights. *Annals of Oncology*.

[10] Makki J. (2015). Diversity of Breast Carcinoma: Histological Subtypes and Clinical Relevance. *Clinical Medicine Insights: Pathology*.

[11] Collin L. J., Yan M., Jiang R. (2019). Oncotype DX Recurrence Score Implications for Disparities in Chemotherapy and Breast Cancer Mortality in Georgia. *NPJ Breast Cancer*.

[12] Guth A. A., Chun Kim J., Schwartz S. (2017). The Relationship of Race, Oncotype DX, and Ki67 in a Population Highly Screened for Breast Cancer. *Breast Journal*.

[13] Toi M., Iwata H., Yamanaka T. (2010). Clinical Significance of the 21-Gene Signature (Oncotype DX) in Hormone -Positive Early Stage Primary Breast Cancer in the Japanese Population. *Cancer*.

[14] Jung J., Hwang K. T., Choi I. S. (2022). Racial Differences in Predictive Value of the 21-Gene Recurrence Score Assay: A Population-Based Study Using the SEER Database. *Breast Cancer*.

[15] Gordon N. P., Lin T. Y., Rau J., Lo J. C. (2019). Aggregation of Asian-American Subgroups Masks Meaningful Differences in Health and Health Risks Among Asian Ethnicities: An Electronic Health Record Based Cohort Study. *BMC Public Health*.

[16] Taparra K., Dee E. C., Dao D., Patel R., Santos P., Chino F. (2022). Disaggregation of Asian American and Pacific Islander Women With Stage 0-II Breast Cancer Unmasks Disparities in Survival and Surgery-to-Radiation Intervals: A National Cancer Database Analysis From 2004 to 2017. *JCO Oncology Practice*.

